# A Stable Micellar Formulation of RAD001 for Intracerebroventricular Delivery and the Treatment of Alzheimer’s Disease and Other Neurological Disorders

**DOI:** 10.3390/ijms242417478

**Published:** 2023-12-14

**Authors:** Laura Gianessi, Alessandro Magini, Roberto Dominici, Stefano Giovagnoli, Diego Dolcetta

**Affiliations:** 1 Department of Pharmaceutical Sciences, University of Perugia, 06123 Perugia, Italystefano.giovagnoli@unipg.it (S.G.); 2 Tuberous Sclerosis Association, 00147 Roma, Italy; 3 Department of Biochemistry, Desio Hospital, ASST-Brianza, 20832 Desio, Italy; 4 Neuroscience Institute of Rosà, 36027 Rosà, Italy

**Keywords:** intracerebroventricular, mTOR, mTOR-I, rapalog, neurological disorders, neurodegeneration, Alzheimer’s disease, DSPE-PEG2000 micelles, micellar liquid formulation, drug stabilisation

## Abstract

A large body of evidence, replicated in many mouse models of Alzheimer’s disease (AD), supports the therapeutic efficacy of the oral mammalian target of rapamycin inhibitors (mTOR-Is). Our preliminary data show that intracerebroventricular (ICV) administration of everolimus (RAD001) soon after clinical onset greatly diminished cognitive impairment and the intracellular beta amyloid and neurofibrillary tangle load. However, RAD001 shows >90% degradation after 7 days in solution at body temperature, thus hampering the development of proper therapeutic regimens for patients. To overcome such a drawback, we developed a stable, liquid formulation of mTOR-Is by loading RAD001 into distearoylphosphatidylethanolamine–polyethylene glycol 2000 (DSPE-PEG2000) micelles using the thin layer evaporation method. The formulation showed efficient encapsulation of RAD001 and a homogeneous colloidal size and stabilised RAD001, with over 95% of activity preserved after 14 days at 37 °C with a total decay only occurring after 98 days. RAD001-loaded DSPE-PEG2000 micelles were unchanged when stored at 4 and 25 °C over the time period investigated. The obtained formulation may represent a suitable platform for expedited clinical translation and effective therapeutic regimens in AD and other neurological diseases.

## 1. Introduction

Taken orally, mTOR-Is (rapamycin and its synthetic analogues, rapalogs) are powerful immunosuppressant drugs that are widely used in the control of organ transplant rejection [1]. They exert their effect on every cell in the body, without distinction, heavily influencing their metabolism and blocking the cell cycle in the G1 phase, thus inhibiting proliferation [2]. The latter action prevents the clonal expansion of lymphocytes, which causes immune system (IS) suppression.

Tuberous sclerosis complex, TSC [3], is a rare inborn disorder caused by mutations in either *TSC1* or *TSC2* genes (both mTOR inhibitors). It is characterised by a hyperactivation of mTOR and benign tumours in almost every organ and therefore also by cerebral subependymal nodules and fast-growing, life-threatening neurinomas [4] called SEGAs (subependymal giant cell astrocytomas). Despite its heavy immunosuppressive effect, and exploiting its antiproliferative one [5], the oral administration of the rapalog everolimus (RAD001) has been the first choice for the treatment of TSC patients since the 2010s. However, the maximum tolerated dose of RAD001 (maximal blood concentration 9–15 ng/mL) can benefit only 40% of patients, reducing the size of SEGAs [6] and improving seizure control [7]. In the same study, a dose that was lower (3–7 ng/mL), albeit tolerable, was completely ineffective. Thus, the efficacy of the treatment seems to be dose-dependent, but the drug displays a narrow therapeutic index.

Under specific conditions, rapalogs exert an immunomodulatory effect. When dendritic cells (DCs) are isolated and cultured in vitro with rapalogs together with an alloantigen and then injected back into the bloodstream, these “rapalog-conditioned” DCs reach the regional lymph nodes (LNs), where they stimulate the production of regulatory T lymphocyte clones (Tregs), inducing tolerance towards that specific alloantigen [8,9,10,11,12,13]. However, until now, this tolerogenic effect has never been exploited through oral administration. In preclinical experiments on mouse models of multiple sclerosis (MS) and experimental autoimmune encephalomyelitis (EAE) [14,15,16,17,18,19], as well as in clinical trials for MS [20] and type 1 diabetes [21], immunosuppressive, antiproliferative effects prevailed, and symptoms reappeared shortly after the end of treatment.

In 2010 [22], a study showed that the oral administration of a high dose of rapamycin for 10 weeks led to memory recovery in cognitively impaired 3xTg Alzheimer’s disease (AD) mice. The 3xTg-AD mouse [23] displays clear cognitive impairment at 6 months of age, with only sporadic beta amyloid (βA) plaques but a significant intracellular βA load and apparent severe synaptic dysfunction. In the above study, autophagy activation played a major role by reducing the intracellular βA and neurofibrillary tangles (NFTs) load and restoring synaptic function. However, the severe immunosuppression caused by the drug [1] may have helped to control the inflammatory components of the disease, which have only recently been described [24]. Prompt cognitive recovery and severe mTOR inhibition has also been demonstrated in other early onset (EOAD) and late-onset Alzheimer’s disease (LOAD) mouse models upon prompt treatment for 13 to 16 weeks after clinical onset [22,25]. With prolonged administration up to 6 months, the amyloid angiopathy was also reduced [26,27]. All models treated with oral rapamycin received a dose of 2.24 mg/kg/24 h for months [28]. Unsurprisingly, the role of immunosuppression on disease recovery was not investigated, with inflammation in AD pathogenesis not being completely understood at the time and the role of autophagy in the disease only just emerging. Again, as in TSC, the low therapeutic index of rapalogs represents the main obstacle preventing immediate transfer to humans, with a dose of a few mg/day being sufficient to induce severe immunosuppression and metabolic side effects [1].

Conversely, due to there being scarce CNS-related side effects (e.g., headache and nausea, which are rarely severe enough to compromise administration), we suggested that local, intracerebroventricular (ICV) administration [29], despite its obvious invasiveness, could promote rapid translation to clinics. Therefore, we administered a RAD001 solution through ICV injection into a 3xTg-AD mouse model using previously assessed experimental conditions [22]. Unexpectedly, the RAD001 ICV administration effectively rescued cognitive functions and mood after a short administration and far beyond the end of treatment [30].

However, our approach was affected by the significant instability of the mTOR-Is tested at body temperature in solution, which rapidly decayed by >90% within a week [30].

To overcome such an important issue, the development of a stable formulation of mTOR-Is was thus required. Such a formulation could help to tailor effective therapeutic regimens (10–15-day treatment cycles) for AD patients. Therefore, the aim of this work was to develop and characterise a micellar delivery platform for RAD001 in vitro that is able to enhance drug stability to overcome the above-mentioned issues that greatly limit its therapeutic potential. For such a purpose, we resorted to a distearoylphosphatidylethanolamine–polyethylene glycol 2000 (DSPE-PEG2000)-based micelle delivery system. This type of micelle is well known for its capacity to entrap and deliver a broad range of hydrophobic drugs [31,32]. A large body of literature addressing DSPE-PEG2000 micelle use in drug delivery exists [33,34,35,36,37]. Properties like a low critical micellar concentration and aggregation number, good stability, a lean preparation process and affordability make DSPE-PEG2000 micelles a potentially good delivery platform even for RAD001. For these reasons, RAD001 was encapsulated in DSPE-PEG2000 micelles and the formulation was characterised in terms of physical–chemical properties and stability over time in conditions relevant to a potential clinical application. If successful, we believe that the sustainability of the materials and processes employed as well as their recognised biocompatibility may promote a relatively fast translation to the clinic.

## 2. Results

In this work, we chose to encapsulate RAD001 into a micellar formulation to overcome the drug stability issues observed in solution that emerged in our previous work, in which its efficacy was demonstrated in AD mice after ICV administration [30].

### 2.1. Validation of the HPLC Method

The HPLC method employed was validated to ensure its suitability and the stability of the formulation. Based on preliminary observations, the method setup was chosen as a compromise between reliability and sensitivity. For this purpose, standard solutions of RAD001 were compared with working solutions that were employed in the stability study. The column and elution conditions were chosen such that only a short analysis time and low material consumption were required.

The results of the method performance are shown in Supplementary Tables S1–S4. The method showed good sensitivity, with a limit of detection (LOD) and limit of quantification (LOQ) of 0.12 ± 0.04 and 0.41 ± 0.02 µg/mL, respectively. Linearity, accuracy, and precision were confirmed by the correlation coefficients always being >0.99 and the lack of statistically significant differences between the standard and working solutions (Supplementary Tables S1 and S2). Likewise, intra-day and inter-day reproducibility was ensured with coefficients of variation below 3% and 5% in most cases for the RAD001 standard and working solutions (Supplementary Table S3). The recovery performances were also good, with values always above 97% in all cases, with, again, no statistically significant difference between standard and working solutions (Supplementary Table S4). The method also had a relatively fast elution time with retention times (Rt) around 9.7 min.

The stability of RAD001 in the formulation was assessed by measuring the amount of RAD001 degradation products. In this regard, since the drug is particularly susceptible to oxidation [38], RAD001 was exposed to highly oxidising conditions and then analysed. The results are shown in Supplementary Figure S1. The chromatogram of the oxidised RAD001 displays multiple peaks between 2 and 4 min, close to the solvent front, ascribable to the more hydrophilic oxidised forms of RAD001. The comparison with the standard profile highlights the ability of the method to discriminate between native and degraded forms of RAD001, as evidenced by the large Rt difference between the corresponding peaks. The chromatograms at 4, 25, and 37 °C recorded over time (Supplementary Figure S2) and the estimation of the overall increase in degradation products overlapped with the RAD001 decay curve at 37 °C in 10% *v*/*v* DMSO/physiological solution (Supplementary Figure S3), providing further evidence of the stability indicating features of the method employed in this work.

### 2.2. Characterisation of RAD001-Loaded Micelles

Based on preliminary observations, 1/10 and 1/20 *w*/*w* RAD001/lipid ratios were chosen to assess the conditions for the optimal loading of RAD001 into micelles. Table 1 shows that the 1/20 ratio ensured nearly complete entrapment of RAD001 in the micelles, while at 1/10, loading efficiency (LE) was almost halved. This result confirms the ability of DSPE-PEG2000 micelles to entrap hydrophobic drugs [31,32]. RAD001 solubility was increased at least 40 times by encapsulation into the micelles. In fact, in line with reports showing a solubility in water, physiological solutions, and buffers of <0.01% [39], drug solubility was around 57 ± 3 µg/mL in physiological solution and 156 ± 9 µg/mL in 10% *v*/*v* DMSO/physiological solution at 4 °C (Supplementary Figure S4). The values increased slightly to 68 ± 5 µg/mL and 181 ± 6 µg/mL, respectively, at 37 °C. When encapsulated in micelles, RAD001 reached concentrations of about 2 mg/mL in the physiological solution at room temperature (25 °C).

The micelles were characterised in terms of size and stability in different storage media, such as 10 mM PBS pH 7.4 and physiological solution.

**Figure 1:**
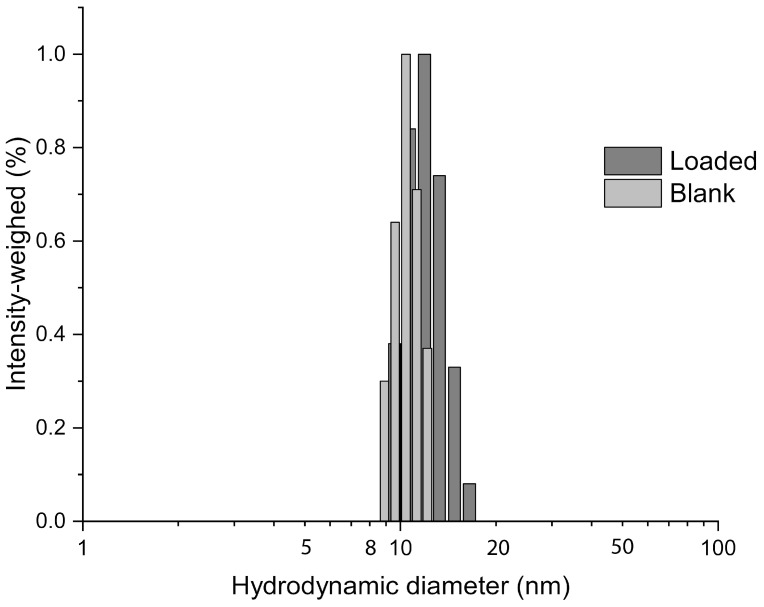
RAD001-loaded micelles show homogeneous and consistent colloidal sizes. Size distributions of Ev-mic obtained by photocorrelation spectroscopy in physiological solution diluted in ultrapure water at 25 °C.

The size of the micelles was consistent with a previous study that found average hydrodynamic diameters between 2 and 35 nm [32]. In our case, the RAD001-loaded micelles were 14 ± 3 nm in size, only slightly larger than the empty micelles (11 ± 2 nm) (Figure 1). This small increase can be ascribed to a loading effect. In addition, the polydispersity was low, between 0.256 and 0.125 for both RAD001-loaded and empty micelles.

**Figure 2:**
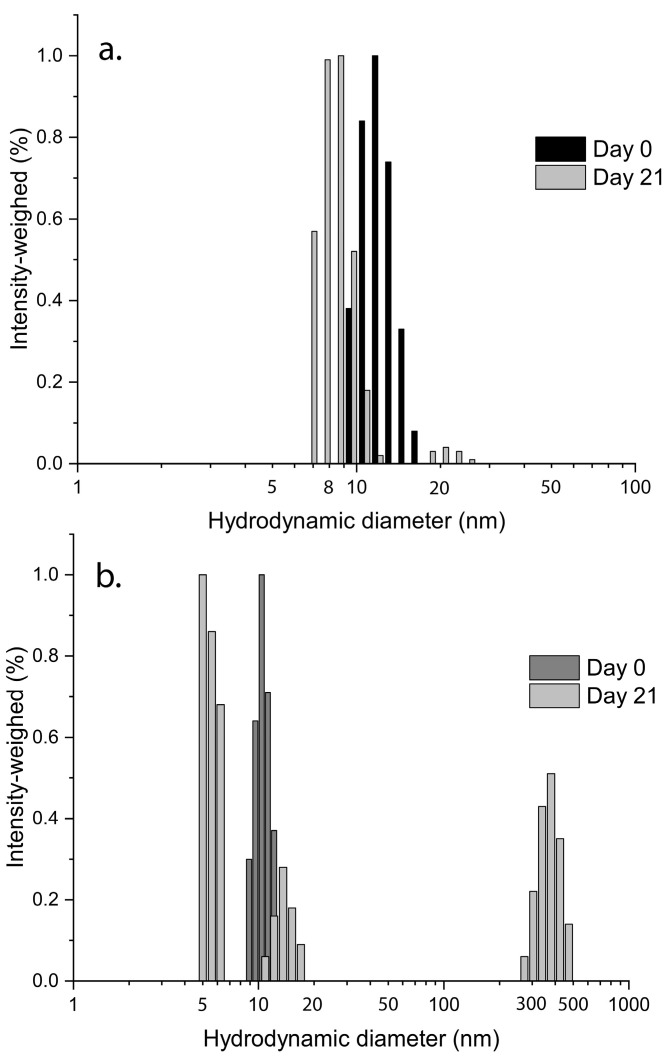
Micelles show higher stability in physiological solution. Size distributions of Ev-mic obtained by photocorrelation spectroscopy in (**a**) physiological solution and (**b**) 10 mM PBS pH 7.4 before and after 21 days at 37 °C.

A short-term analysis of the stability of the loaded micelles was performed at 37 °C over 21 days in two different storage media (Figure 2). The micelles were more stable in the physiological solution as the size distribution was nearly unchanged with a non-significant decrease in terms of mean diameter on day 21. In PBS, new peaks appeared at around 5 nm and 450 nm. This change suggests a loss of the micelle structure as well as aggregation with a possible release of the drug, which may also aggregate over time into larger clusters. As a result, RAD001-loaded micelles seemed to be less stable if they were incubated in PBS.

**Figure 3:**
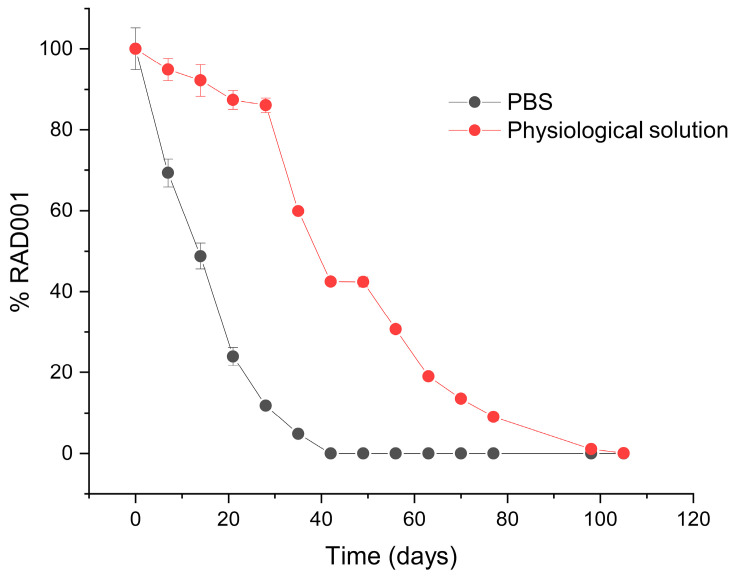
RAD001 loaded in micelles shows higher stability in physiological solution. RAD001 stability in micelles in 10 mM PBS pH 7.4 and physiological solution over time at 37 °C.

To support this observation, the micelle preparations were assayed by means of HPLC in the same conditions over more than 100 days of incubation. The obtained profiles confirmed the decay of RAD001 in PBS that started earlier than in the physiological solution (Figure 3). RAD001 was completely lost after 40 days in PBS, while in the physiological solution complete degradation was only recorded after 100 days. This matches the assumption that the drug is progressively released early and then slowly degraded in PBS. DSPE-PEG2000 micelles can be significantly perturbed by strong electrolytes. Such events are influenced by factors like lipid and ion concentrations. In particular, size increases and critical micellar concentrations decrease with increasing ionic strength due to a stabilisation of negatively charged phosphate groups by counterions in buffered or saline media [36,40].

In our conditions, micelle destabilisation in PBS may occur through a perturbation of the surface potential due the additional ions and thus increasing the tendency to aggregate and the subsequent structural changes that may lead to drug leakage. The elucidation of this aspect is far from the aim of this work and will require further evaluation in future studies. Overall, our study suggests that physiological solution is the preferred storage medium for the RAD001-loaded micelles.

### 2.3. Evaluation of Ev-Sol and Ev-Mic Stability

**Figure 4:**
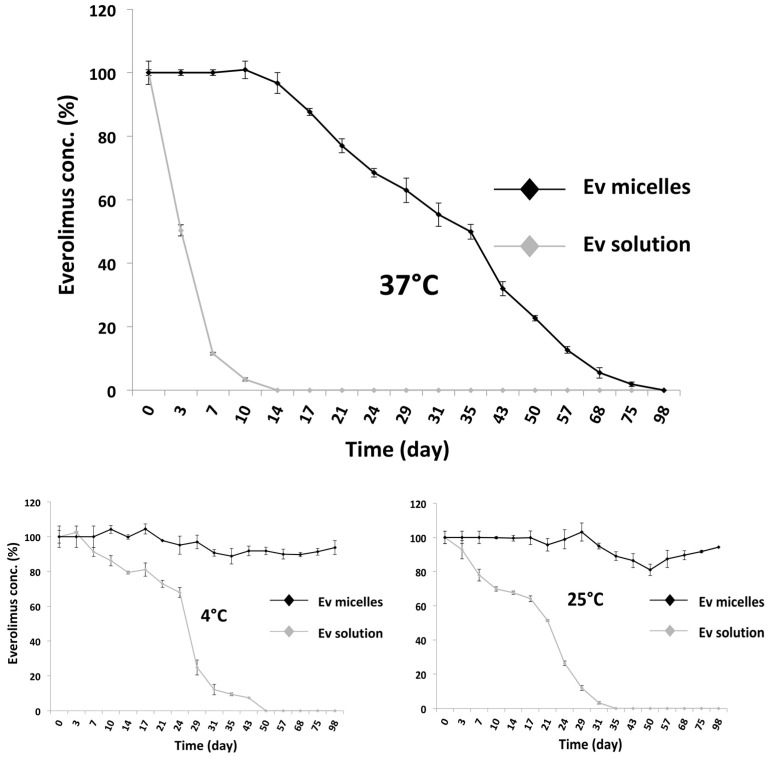
Micelles stabilise RAD001 in solution. RAD001 decay in 10% *v*/*v* DMSO/physiological solution (vehicle) (grey) and in micelles (black) at 37 °C, 25 °C, and 4 °C. After 15 days at 37 °C, over 95% of the Ev-mic activity was still maintained, while Ev-sol decayed completely.

To evaluate the stability of the RAD001 in micelles (Ev-mic), samples were incubated at 4 °C, 25 °C, and 37 °C, and compared with RAD001 solution in 10% *v*/*v* DMSO/physiological solution at established time points, by HPLC-UV analysis (Figure 4). At 37 °C. Ev-mic showed prolonged stability that was far superior to that of Ev-sol, as reported in Figure 4A; while Ev-sol dropped to zero at day 14, Ev-mic was undetectable by day 98. As shown in Figure 4B,C, Ev-mic was more stable at 25 °C and 4 °C compared to Ev-sol and retaining around 100% of the drug concentration after 98 days of incubation, while Ev-sol decayed completely at day 35 at 25 °C and at day 50 at 4 °C.

### 2.4. Toxicity of Empty Micelles

**Figure 5:**
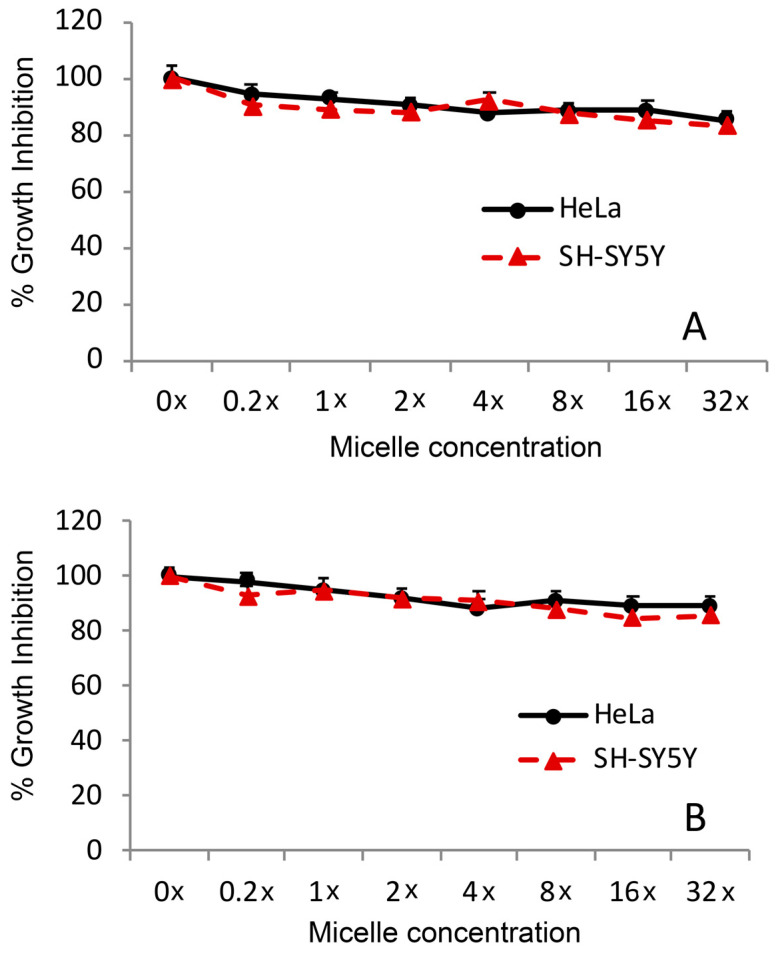
Micelle safety in vitro. HeLa and SH-SY5Y cells were challenged for (**A**) 24 h and (**B**) 48 h with increasing concentrations of blank DSPE-PEG2000 micelles and analysed using the MTT (3-(4,5-dimethylthiazol-2-yl)-2,5-diphenyl-2H-tetrazolium bromide) assay. Cells were seeded in a 96-well plate at concentrations of 3000 or 6000 cells/well, respectively, and were maintained in culture medium (100 µL) for 24 h before the treatment.

The assay was conducted using HeLa and SH-SY5Y cells, which were seeded in a 96-well plate at a concentrations of 3000 and 6000 cells/well, respectively, and treated for 24 and 48 h. These two cell lines were chosen as both are well established models that can be used to evaluate drug toxicity [41,42] and pharmacological effects [43,44]. In all of the cellular experiments, we maintained the same difference in cell concentration, taking into account the different replication rates between the HeLa and SH-SY5Y cells. Empty micelles, suspended in PBS or in physiological solution, were used at final concentration of 0.2, 1, 2, 4, 8, 16, and 32× relative to the Ev micelle concentration of 5 nM (considered as 1×) (Figure 5).

Cell proliferation was nearly unchanged after empty micelle treatment. The same behaviour was observed in PBS (Supplementary Figure S5).

### 2.5. Evaluation of Ev-Sol and Ev-Mic Stability Using Cell Cultures

**Figure 6:**
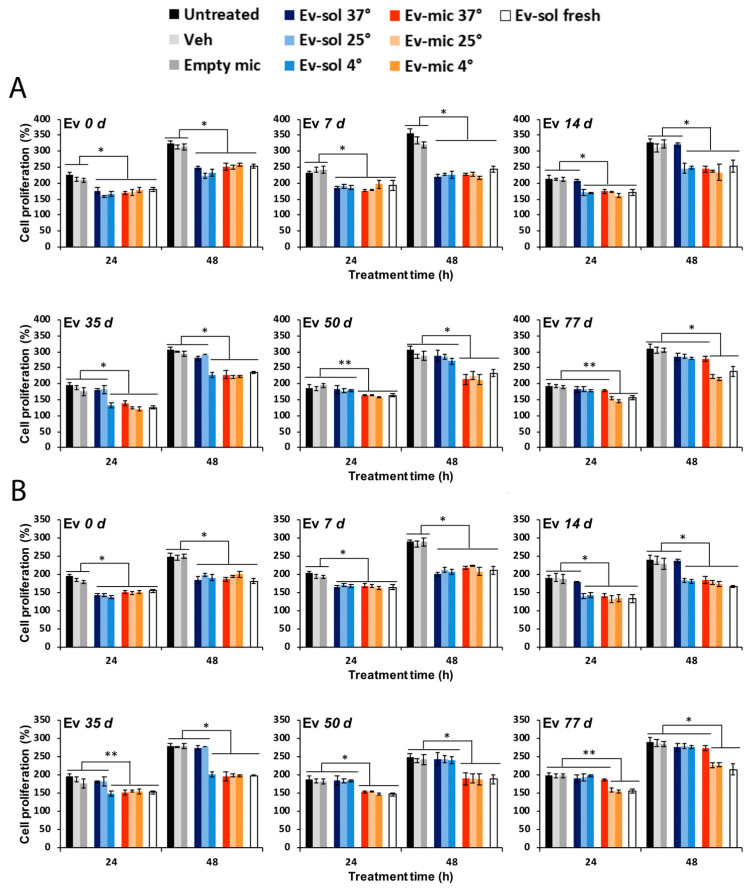
Micelles extend the activity of RAD001 in vitro. Ev-sol and Ev-mic activity on (**A**) HeLa and (**B**) SH-SY5Y cells was measured using the MTT assay. HeLa and SH-SY5Y cells were seeded into a 96-well plate at concentrations of 3000 cells/well and 6000 cells/well, respectively, and were maintained in culture medium (100 µL) for 24 h before the treatment. Ev-sol and Ev-mic were used fresh and after incubation at 37 °C, 25 °C, and 4 °C for 7, 14, 35, 50, and 77 days. Untreated, DMSO-treated (Veh), and blank micelle-treated (Empty mic) cells were employed as negative controls, while fresh RAD001 solution-treated cells (Ev-sol fresh) were used as positive controls. All treatments were performed at the same equivalent concentration of RAD001 (5 nM). Data are representative of three independent experiments. * *p* < 0.001, ** *p* < 0.01.

To verify the greater stability of Ev-mic indicated by the HPLC analysis, Ev-sol and Ev-mic activity was assessed using HeLa and SH-SY5Y cells (Figure 6A,B). Ev-sol and Ev-mic, prepared in the same manner as in the stability experiment, were used at a final concentration of 5 nM in fresh medium or after incubation at 37 °C, 25 °C, and 4 °C for 7, 14, 35, 50, and 77 days. The RAD001 IC50 is 1.6–2.4 nM [45], so a 5 nM concentration seemed suitable for assessing its inhibitory activity while avoiding toxicity. The inhibition of cell proliferation was evaluated using an MTT (3-(4,5-dimethylthiazol-2-yl)-2,5-diphenyl-2H-tetrazolium bromide) assay. Untreated, DMSO/physiological solution-treated (Veh), and empty micelle-treated (Empty mic) cells were used as negative controls. Fresh EV-sol was used as positive control. The day after the MTT treatment, cell proliferation was measured by spectrophotometric readings at 589 nm and 650 nm. As reported in Figure 6A, at 37 °C, the Ev-mic activity was no longer detectable after 77 days, while at 25 °C and 4 °C, it remained mostly unchanged. It lasted a bit longer at 25 °C and 4 °C. Ev-sol activity ceased at 35 and 50 days, respectively. The results obtained using the HeLa cell line were confirmed by the analysis conducted on the SH-SY5Y cells using the same experimental conditions (Figure 6B). At 37 °C, the Ev-sol activity was not detectable on the 14th day. Therefore, consistent with the observed stabilisation of RAD001, these results demonstrated a prolonged pharmacological activity of Ev-mic compared to Ev-sol in cell cultures.

## 3. Discussion

There is evidence demonstrating that a 10-to-16-week (or longer) oral administration of rapamycin is efficacious in the recovery from cognitive decline in AD mouse models, and it is beneficial in both EOAD and LOAD [22,25,26,27,30]. However, this result was obtained by administering the drug orally at a daily dose of 2.24 mg/kg [28], which is a high dosage compared to standard rapamycin therapies.

More than a decade after the first publications demonstrating its efficacy in mice, a phase 1 clinical trial (NCT04200911, CARPE DIEM, https://clinicaltrials.gov/study/NCT04200911, accessed on 12 August 2023) for oral rapamycin on already symptomatic patients has been completed and a phase 2 study (NCT04629495, REACH, https://clinicaltrials.gov/study/NCT04629495, accessed on 12 August 2023) is now in progress. Therefore, the application of rapalogs to neurological disease treatment has a strong supporting rationale; however, we believe that the systemic route may not be the best choice to ensure long-term therapeutic effects and adherence. Even though it is hampered by unarguable invasiveness, based on our preclinical observations, ICV administration may provide an alternative and valuable modality to greatly enhance the efficacy and safety of treatments.

In our previous work [30], ICV administration of RAD001 increased the cerebral drug concentration while maintaining low blood levels, and therefore reducing systemic immunosuppression. Our results after 2 weeks of treatment matched those observed in previous works after 10–16 weeks [22,25,26,27].

An elegant paper by Mohammad et al. [46] on the mechanism of action of fingolimod, a widely used drug for MS control, may provide a reasonable explanation for our results. They demonstrated that, in mice, there is a continuous flow of DCs patrolling the brain. DCs enter the brain through choroid plexuses, then cross the ependymal layer, proceed along the rostral migratory stream, and exit the brain through the *lamina cribrosa*, reaching the lymphatic vessels and then the cervical LNs. Fingolimod exerts its therapeutic effect (both in mice and in humans) by blocking DC egression from the brain, preventing them from reaching the cervical LNs, bringing inflammatory signals and relapse. This results in DCs gathering in the olfactory bulb.

**Figure 7:**
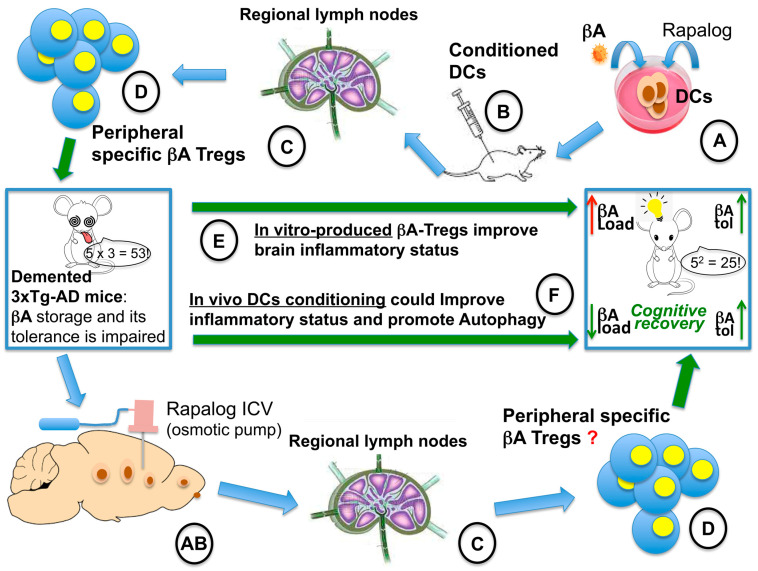
Hypothetical explanation of the prolonged recovery observed in the AD mouse model treated with ICV everolimus [30]. On the left, a cognitively impaired 3xTg-AD mouse. On the right, a cognitively recovered 3xTg-AD mouse. On the upper part of the figure, the in vitro production of βA-specific Tregs is described: (**A**) in vitro conditioning of DCs with βA and a rapalog [8,9,10,11,12,13], (**B**) injection of conditioned DCs into the bloodstream, (**C**) conditioned DCs migrate to regional lymph nodes; (**D**) infusion into the bloodstream of specific Tregs into 3xTg-AD mouse; (**E**) the infusion of βA-specific Tregs leads to improved βA tolerance and cognitive recovery [46] (green arrow), but not to βA load reduction (red arrow). On the bottom part of the figure, the in vivo approach and the hypothetical explanation of its rapid and prolonged efficacy are described [30,47]. (**AB**) The rapalog-loaded osmotic pump is implanted into the lateral ventricle, likely leading to conditioning of CNS-patrolling DCs; (**C**) conditioned DCs migrate to regional lymph nodes; (**D**) the treatment might lead to in vivo βA-specific Treg production that could lead to both βA tolerance [46] (green arrow) and to reduction of the βA load, promoting autophagy (**F**). The combined effect could explain the results observed.

It has been recently demonstrated that the administration of βA-specific Tregs, obtained in vitro, ameliorates both the inflammatory pathology and cognitive impairment in 3XTg-AD mouse model [47]. In our case [30], a similar effect may be speculated to occur due to a strong conditioning of the DCs by the locally administered RAD001. It is possible that “RAD001-conditioned” DCs, after gathering and processing self-antigens (βA), migrated to the regional LNs, where they found an active immune system that induced the expansion of specific Treg clones. Unlike oral administration, in which immune cells are blocked in the G1 phase of the cell cycle [2], we might have replicated in vivo and locally in the CNS, the “conditioning” of DCs by RAD001, which, so far, has been only reported in vitro [8,9,10,11,12,13]. Together with autophagy-linked βA depletion, this might help to control the inflammation in AD [24,48,49,50] and restore the impaired tolerance to beta-amyloid (Figure 7).

In this context, prolonging the treatment period could promote the improvement of the therapeutic outcome. Unfortunately, the evident instability of rapalogs in solution strongly limits the possibility of achieving long-term effects and establishing a suitable therapeutic regimen. Therefore, stabilisation approaches that can significantly extend the lifespan of rapalogs in liquid formulations, either in the body or upon storage, can drive the development of successful clinical applications.

The choice of micellar formulations depends not only on their drug preservation capacity but also on the ease of preparation, storage, and handling. These features have enabled micellar formulations to reach phase 3 trials with high chances of market approval [51,52].

Micelles are already clinically tested to deliver anticancer drugs [53]. In particular, DSPE-PEG2000 micelles have been found to be effective in delivering a broad range of drugs in animal models [54,55,56,57] and RAD001 in vitro [58]. Properties such as a low critical micellar concentration as well as small size increase the appeal of DSPE-PEG2000 micelles since they can enable effective drug accumulation at the site of action [31,32]. Moreover, as also proven for RAD001 in this work, DSPE-PEG2000 micelles ensure nearly complete entrapment of hydrophobic drugs in their core with optimal production yields.

In our case, the obtained RAD001 micelles showed a suitable size and loading capacity and could stabilise RAD001 over an extended period of time which is comparable to long-term storage and prolonged therapeutic regimens. Moreover, the in vitro experiments on HeLa and SH-SY5Y cell lines confirmed the biocompatibility of DSPE-PEG2000 (Figure 5) as well as the stabilisation of RAD001 that correlated with the observed RAD001 decay over time (Figure 6). These results encourage the further development of this strategy especially when considering the translational potential of the formulation. In this regard, it must be stressed that the approach in this work, which influenced the choice of materials and processes, was to keep the system as simple as possible in order to limit potential bottlenecks in the transfer to the clinic.

Our results suggest that ICV administration of the RAD001-loaded DSPE-PEG2000 micelles may extend RAD001’s therapeutic effect compared to the solution, likely improving the impact of the mTOR inhibition on patients’ quality of life and reducing the immunosuppression and metabolic side effects.

Although invasive, ICV administration requires a minor surgical intervention, and it is currently in clinical use to treat rare conditions [59,60,61,62]. Bacterial meningoencephalitis that is sensitive to antibiotics that are unable to cross the blood–brain barrier and primary cerebral lymphomas are just two examples of conditions currently being treated using ICV therapy. Possible complications include local infection and intracerebral haemorrhage; however, these complications only result in a temporary therapy interruption without serious long-term effects [61].

Nevertheless, the use of ICV administration should be based on a careful risk–benefit evaluation of the patients’ profile. In fact, this procedure can have a greater economic impact on national health systems than more conventional routes of administration, even though we believe that proper automation may result in minimal-risk operations that can be performed by a general surgeon.

Through ICV administration, RAD001-loaded micelles may represent an immediately available treatment for several forms of dementia and neuroproteinopathies [63]. This consideration may also apply to TSC for which the oral administration of rapalogs is already the treatment of choice. Unfortunately, 60% of patients fail to obtain control of SEGA growth and seizures. Studies on patient cohorts suggest that rapalogs’ treatment effect in TSC could be dose-dependent [6,7]. Therefore, local, high doses through ICV administration might considerably improve therapy outcomes.

A recently proposed alternative strategy to ICV administration consists of a combination of a selective peripheral rapamycin inhibitor (Rapablock) and a third-generation mTOR-I that is able cross the blood-brain barrier (BBB) (Rapalink-1) [64]. This so-called “Shokat combination” is able to ensure the pharmacological effects on the CNS while avoiding peripheral side effects. Nevertheless, this strategy may fail in some cases, such as progressive autoimmune diseases, some forms of AD [65], and brain tumours [66], where inflammation is known to alter the integrity of the BBB. In such cases, the inhibitor Rapablock can cross the BBB and inhibit Rapalink-1 within the brain.

## 4. Materials and Methods

### 4.1. Preparation of RAD001 Stock, Standard, and Working Solutions

Everolimus (RAD001, Selleck Chemicals, Houston, TX, USA) stock solutions were prepared by dissolving the drug in methanol (VWR, Milan, Italy) at a concentration of 1 mg/mL. Standard solutions at 10, 5, 2.5, 1.25, 0.625 µg/mL were obtained by diluting the RAD001 stock solution in methanol. Working solutions of RAD001 were obtained by diluting the stock solutions to a concentration of 10 µg/mL (Ev-sol) in a 10% *v*/*v* DMSO/physiological solution. RAD001-loaded micelle (Ev-mic) working solutions were prepared using the RAD001 extraction method described below. All solutions were prepared using ultrapure water obtained by reverse osmosis (resistivity 18.2 MΩ·cm at 25 °C; total organic carbon ≤ 5 ppb, Milli-Q purification apparatus, Millipore, Bedford, MA, USA).

### 4.2. Preparation of RAD001-Loaded Micelles

RAD001-loaded DSPE-PEG2000 (Lipoid, Steinhausen, Switzerland) micelles were prepared by employing the thin layer evaporation method. Briefly, carefully weighed amounts of RAD001 and lipids were dissolved in 3 mL of chloroform (Sigma-Aldrich, Milan, Italy) in a 50 mL round flask at 1:10 and 1:20 *w*/*w* ratios. The solvent was removed by gentle evaporation at room temperature (RT) under nitrogen stream and vacuum dried for 1 h to eliminate any residual solvent traces. The thin film obtained was reconstituted in an exact volume (3 mL) of physiological solution by slowly adding the solvent and vortexing. After complete reconstitution, a clear micellar suspension (Ev-mic) was obtained and was stored at 4 °C until use.

### 4.3. HPLC Method Validation and RAD001 Quantification

#### 4.3.1. HPLC Method Validation

The HPLC method employed in this work was assessed following the International Conference of Harmonization (ICH) guidelines. Linearity, precision, accuracy, recovery, limit of detection (LOD), and limit of quantification (LOQ) were assessed in accordance with recommendations [67,68,69]. The method was also tested for its capacity to detect RAD001 degradation products. For this purpose, RAD001 was oxidised by exposure to a 30% *v*/*v* H_2_O_2_ solution for 24 h at 37 °C and then analysed by HPLC [68].

The HPLC instrument was a Portlab STAYER HPLC system equipped with a UV detector, parallel pump, and Triathlon autosampler (Portlab, Rome, Italy). The HPLC conditions were based on a published work with some modifications [70]: isocratic mode with acetonitrile/water (60:40, *v*/*v*), eluted at 0.8 mL/min, 15 µL injection volume, Zorbax C8, 4.6 mm ID × 250 mm, 5 μm column (Agilent, Milan, Italy) equilibrated at 55 °C. UV detection was performed at 278 nm. The RAD001 calibration curves were generated using dilutions of stock solutions, which were prepared as described above. All measurements were performed in triplicate.

#### 4.3.2. RAD001 Quantification

The drug was quantified using the validated HPLC method. While Ev-sol working solutions were directly subjected to analysis without further dilution, Ev-mic samples were prepared by extracting RAD001 micelle formulations stored in the physiological solution. A 200-fold direct dilution of the suspensions with a 1:1 DMSO/chloroform mixture was performed before the HPLC analysis. To reduce carry-over effects and the possible accumulation of residual lipids in the column, Ev-sol and Ev-mic samples were injected in an alternating fashion, followed by periodic washing of the column with chloroform and methanol. All measurements were performed in triplicate and the results are expressed as mean ± S.D.

#### 4.3.3. RAD001 Solubility and Micelle Loading Efficiency

RAD001 solubility was assessed in the physiological solution and 10% *v*/*v* DMSO/physiological solution by incubating 2 mg/mL RAD001 suspensions at 4 °C and 37 °C for 2 h.

The RAD001 loaded into the micelles was measured by determining the residual undissolved and/or non-entrapped RAD001 after hydration. For this purpose, loaded micelle suspensions were ultracentrifuged at 50,000 rpm for 30 min at 4 °C using an Optima TL ultracentrifuge equipped with a TLA-100.4 rotor (Beckman, Palo Alto, CA, USA) and aliquots of the supernatants were diluted in DMSO and then analysed as described above. The loading efficiency (*LE*) was calculated as follows:

(1) $$LE\left(\%\right)=\frac{Amount\;of\;RAD001\;in\;the\;centrifuged\;micelle\;suspension}{Amount\;of\;RAD001\;added}\times 100$$

A further check to exclude the presence of suspended particulates was performed by photocorrelation spectroscopy, as described below, to detect aggregates. All measurements were performed in triplicate and the results are expressed as mean ± S.D.

### 4.4. Micelle Size

The size of Ev-mic and blank micelles was assessed by photocorrelation spectroscopy. In brief, the samples were diluted with ultrapure water and analysed at 25 °C and the hydrodynamic diameter was determined using a Nicomp 380 ZLS photocorrelator (PSS, Santa Barbara, CA, USA) equipped with a 35 mW He/Ne laser (=654 nm) and an APD detector.

### 4.5. Micelle Stability

Loaded RAD001 stability was evaluated in the physiological solution and 10 mM phosphate-buffered saline (PBS, Sigma-Aldrich, Milan, Italy), pH 7.4, as storage solutions. The samples were incubated at 37 °C over 21 days and measurements were performed by withdrawing 100 µL aliquots at established time intervals. These were diluted in ultrapure water at RT and analysed. Changes in micelle size were monitored by photocorrelation spectroscopy. All measurements were performed in triplicate and the results are expressed as mean ± S.D. The RAD001 stability in micelles was assessed by HPLC as described above. The Ev-sol working solutions and Ev-mic suspensions in the physiological solution were incubated at 4, 25, and 37 °C over time and the RAD001 decay was followed until complete degradation was achieved. All measurements were performed at least in triplicate and the results are expressed as mean ± S.D.

### 4.6. Cell Cultures

Both HeLa cells (uterine cervix carcinoma cell line) and SH-SY5Y cells (neuroblastoma cell line) were obtained from the American Type Culture Collection (Manassas, VA, USA) and were cultured in Dulbecco’s modified Eagle’s medium (DMEM with L-glutamine) supplemented with 10% (*v*/*v*) heat-inactivated foetal bovine serum (FBS), 100 U penicillin, and 100 U streptomycin in a humidified incubator with 5% CO_2_ at 37 °C.

### 4.7. Micelle Cytotoxicity Test

The toxicity of empty micelles was evaluated using HeLa and SH-SY5Y cells and the MTT assay. The cells were seeded into a 96-well plate at concentrations of 3000 and 6000 cells/well, respectively, and maintained in 100 µL of medium. The assay was performed at 24 and 48 h after treatment. Empty micelles, suspended in PBS or in the physiological solution, were used at a final concentration of 0.2, 1, 2, 4, 8, 16, or 32×, where 1× is equivalent to the Ev-mic RAD001 concentration of 5 nM.

### 4.8. Functional Stability of Incubated RAD001 Micelles In Vitro

The stability of the RAD001 solution (Ev-sol) and RAD001-loaded micelles (Ev-mic) was determined by evaluating cell proliferation after treatment. Briefly, HeLa and SH-SY5Y cells were seeded into a 96-well plate at concentrations of 3000 cells/well and 6000 cells/well, respectively, and were maintained in culture medium (100 µL) for 24 h before the treatment. Every condition was tested in triplicate. Ev-sol and Ev-mic were suspended in cell culture medium at a final concentration of 5 nM and were used fresh or after incubation at 37 °C, 25 °C, and 4 °C for 7, 14, 35, 50, and 77 days. As controls, untreated, DMSO-treated (Veh), and empty micelle-treated (Empty mic) cells were assayed. Cell proliferation was evaluated using the 3-[4,5-dimethylthiazol-2-yl]-2,5-diphenyltetrazolium bromide (MTT; Sigma-Aldrich) assay according to the manufacturer’s instructions. The cells were mixed with 10 µL MTT (0.5 mg/mL) and maintained for 4 h in a humidified incubator at 37 °C. Then, 100 µL of solubilisation solution (10% SDS with 0.01 N HCl) was added to each well. The solubilisation of formazan crystals was performed overnight in an incubator at 37 °C. The following day, cell proliferation was evaluated by a Beckman Coulter DTX 880 Multimode Detector (Beckman Coulter Inc., Brea, CA, USA) using a 589 nm line and 650 nm reference.

### 4.9. Statistics

Student’s *t*-test and one- or two-way ANOVA with a Bonferroni post hoc test was used to determine statistical significance. Significance was defined as *p* < 0.05. The data are pooled results (mean ± SEM) or representative images from three experiments.

## 5. Conclusions

The RAD001 entrapment in DSPE-PEG2000 micelles resulted a successful stabilisation strategy. This approach was able to produce a remarkable improvement in RAD001 stability that may promote the development of effective long-term treatment regimens for certain neurological disorders. Previous evidence of the therapeutic potential of the local administration of rapalogs [30], combined with the suitable features and high translatability of the proposed technology, support the development of a therapeutic approach based on continuous ICV infusion of mTOR-Is. Further steps have been planned to address the therapeutic potential of our strategy in proper preclinical models.

## Figures and Tables

**Table 1:** Loading efficiency (LE) of DSPE-PEG2000 micelles at different RAD001 fractions in physiological solution.

RADD001/Lipid *w*/*w* Ratio	RADD001 Molar Fraction	LE (%*w*/*w*) ± S.D. (n = 3)
1/10	0.29	56.4 ± 4.5
1/20	0.14	98.7 ± 2.3

## Data Availability

The data presented in this study are available on request from the corresponding author and prof Stefano Giovagnoli.

## Supplementary Materials

The following supporting information can be downloaded at: https://www.mdpi.com/article/10.3390/ijms242417478/s1.
