# Supplementary material for: A Stable Micellar Formulation of RAD001 for Intracerebroventricular Delivery and the Treatment of Alzheimer’s Disease and Other Neurological Disorders
Source: Int J Mol Sci. 2023 Dec 14;24(24):17478. doi: 10.3390/ijms242417478 (PMC10744130; doi:10.3390/ijms242417478)
Supplement: Supplementary file 1 [file ijms-24-17478-s001.zip › Supplementary figures.pdf]

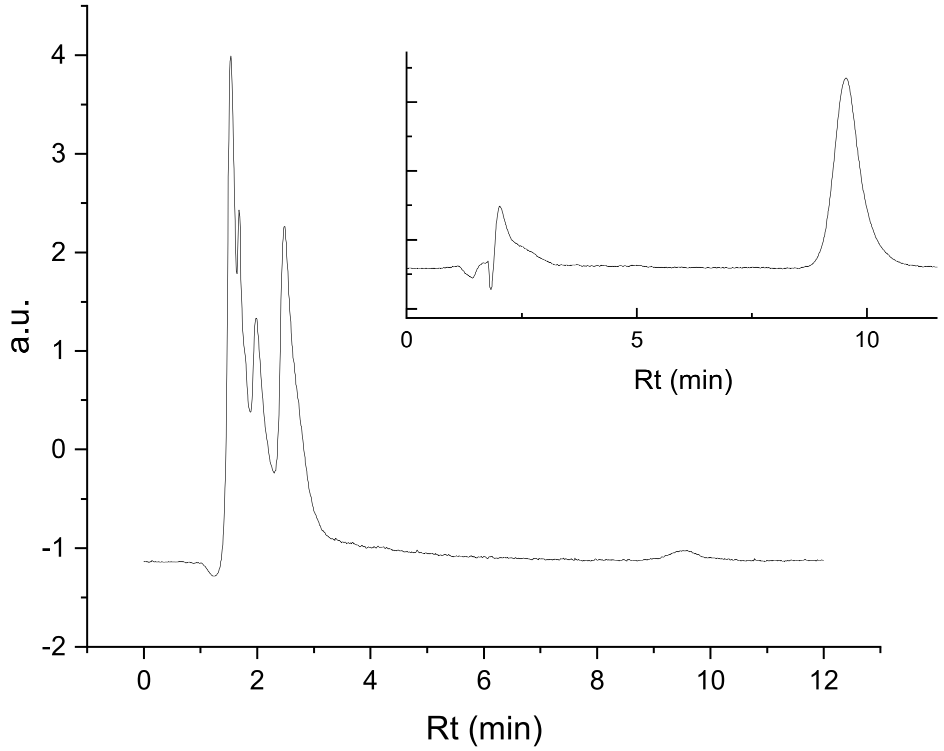

Figure S1- HPLC profile of oxidized RAAD001. RAD001 was exposed to 30% H_2O_2 for 24 hours at 37°C. The insert shows the standard RAD001 chromatogram.

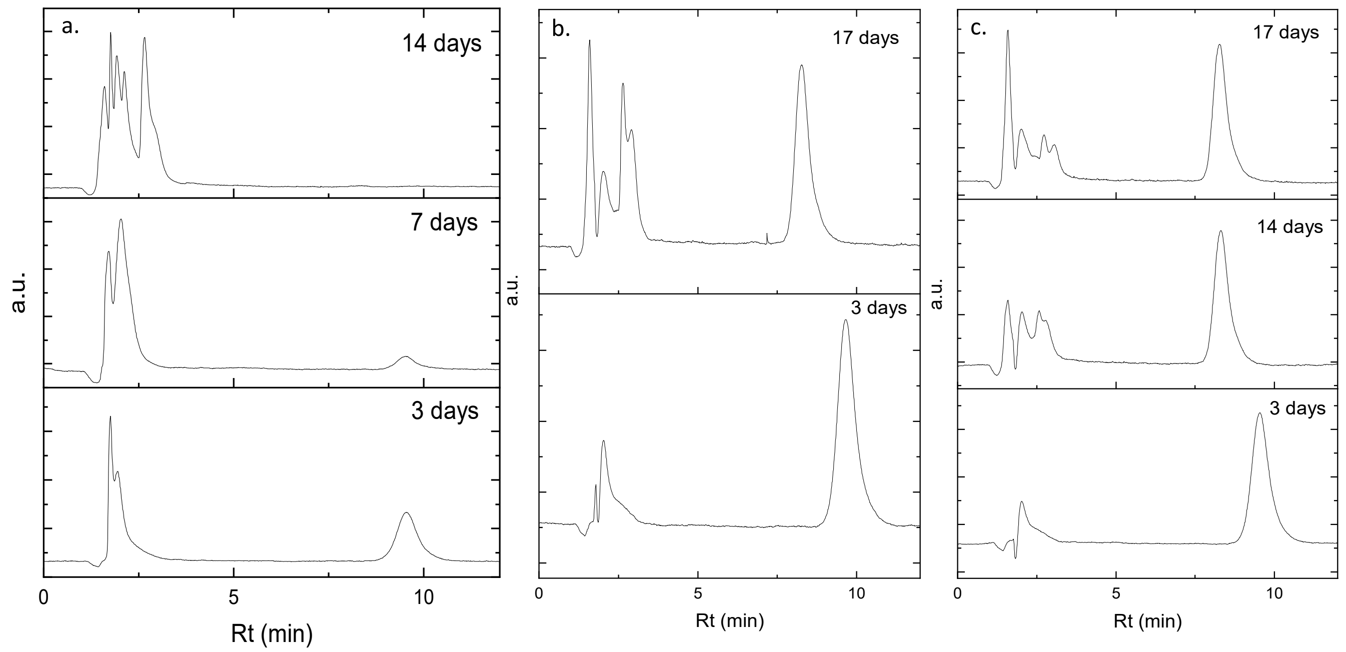

Figure S2- HPLC profiles over time of the RAD001 solutions incubated at a. 37°C, b. 25 °C, c. 4 °C.

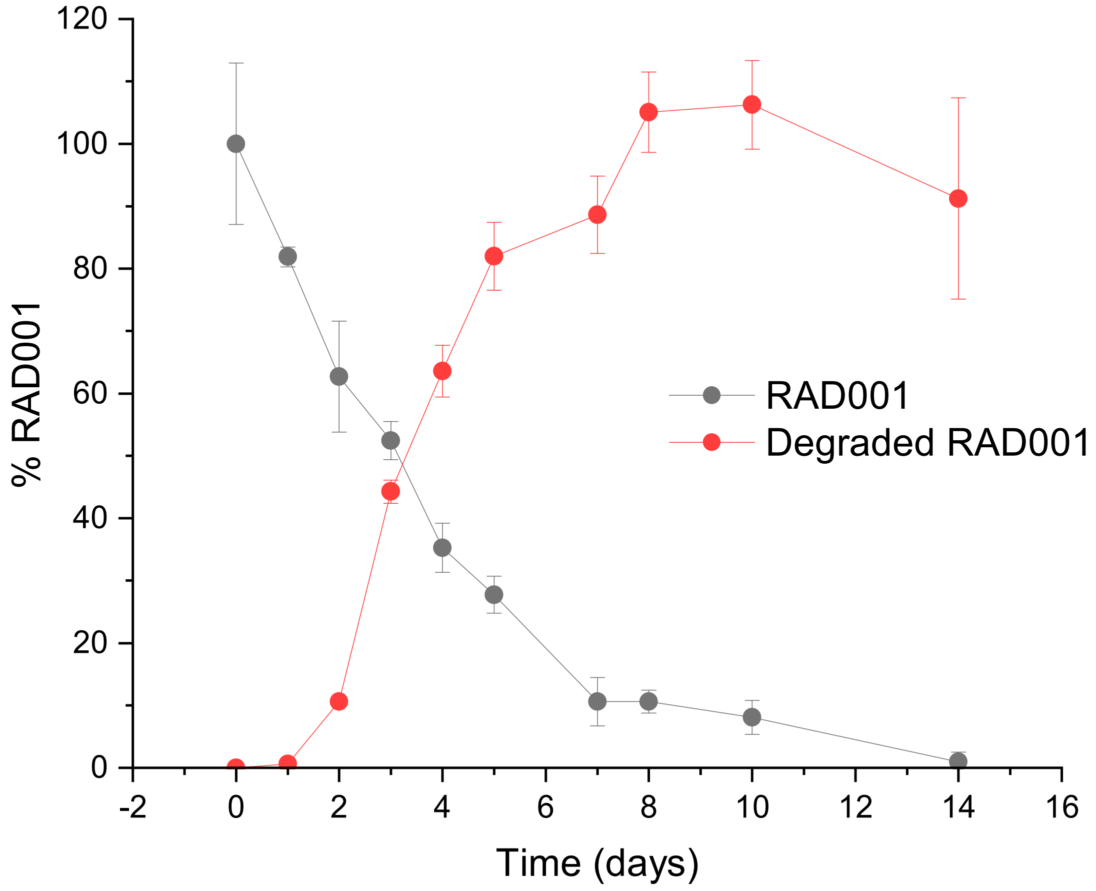

Figure S3- RAD001 decay profile in 10%v/v DMSO/physiologic solution in comparison with an overall estimation of degraded RAAD001 growth over time at 37 °C.

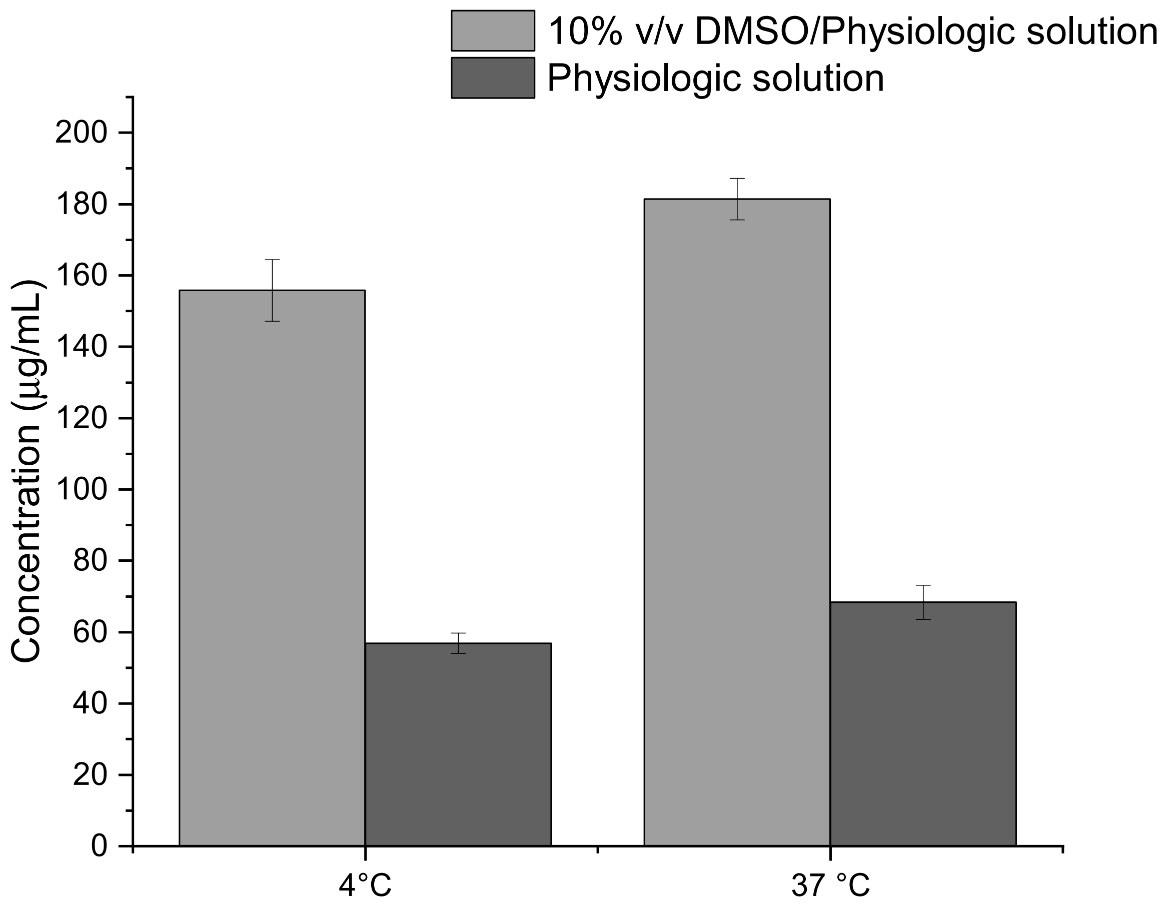

Figure S4- Solubility of RAD001 in 10% v/v DMSO/physiologic solutions and physiologic solution at 4 and 37°C. The analysis was performed after incubation of a 2 mg/mL RAD001 suspension for 2 hours.

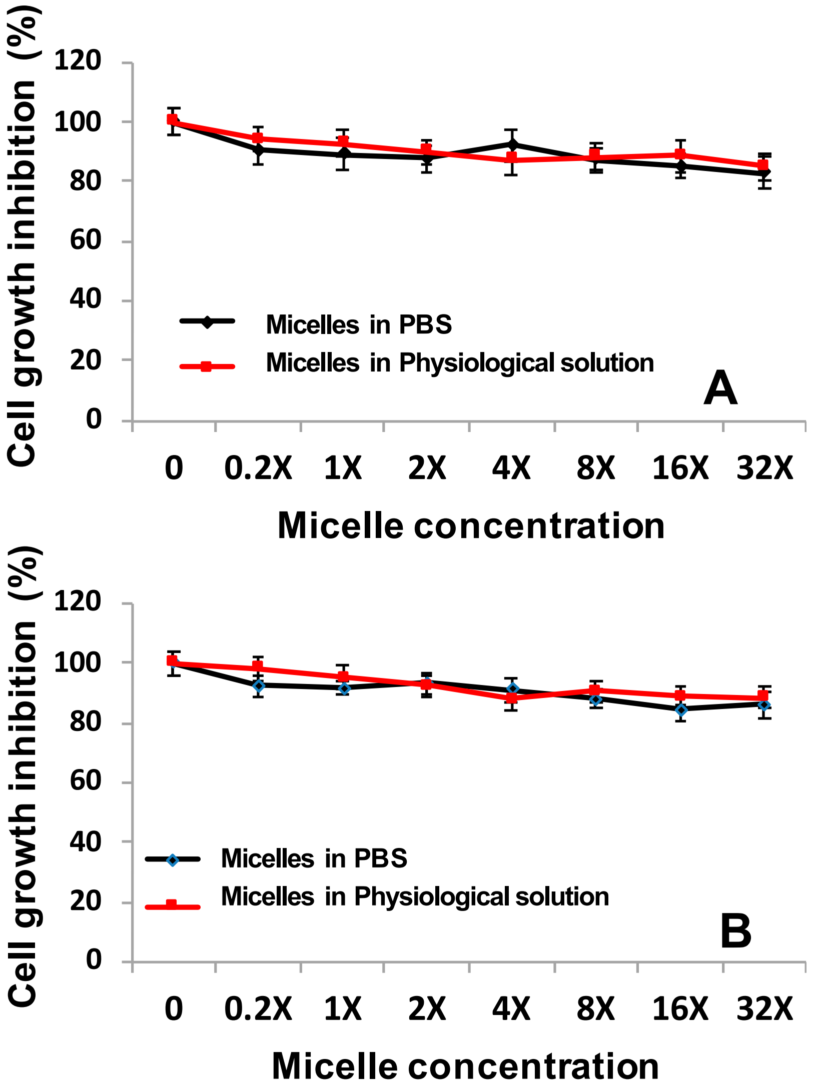

Figure S5- HeLa cells were challenged for (A) 24 h and (B) 48 h with increasing scalar concentration of blank DSPE-PEG2000 micelles stored in PBS and physiological solution and analysed by the MTT assay. Cells were seeded in a 96-well plate at the concentration of 6000 or 3000 cells/well, respectively, and were maintained in culture medium (100 μl) 24 h before the treatment.
