# Supplementary material for: A Stable Micellar Formulation of RAD001 for Intracerebroventricular Delivery and the Treatment of Alzheimer’s Disease and Other Neurological Disorders
Source: Int J Mol Sci. 2023 Dec 14;24(24):17478. doi: 10.3390/ijms242417478 (PMC10744130; doi:10.3390/ijms242417478)
Supplement: Supplementary file 1 [file ijms-24-17478-s001.zip › Supplementary Tables.pdf]

Table S1- Statistics of standard solutions.

Concentration (µg/mL)	%C.I.* ($p<0.05$, n=4)	% Mean accuracy	% C.V.*
**Standard solutions**			
0.625	±5.1	105.1	3.3
1.125	±4.4	98.5	2.7
2.5	±1.8	100.3	1.9
5	±2.7	103.2	2.9
10	±1.1	101.4	1.6

* %C.I., % confidence interval, C.V., coefficient of variation.

Table S2- Linear regression curve statistics for standard solutions, Ev-sol and Ev-mic working solutions.

	Range (µg/mL)	n	Terms	Regression curves				
				Coefficient	Standard Error	r^2	t*	p* (α=0.05)
**Standard solutions**	0.625-10	5	**A***	2.5347	0.0000655	0.9992	82.022	<0.0001
			**B***	10.341	0.00411		-1.698	0.132
**Ev-sol control solutions**	0.625-10	5	**A***	2.3788	0.000563	0.9961	11.786	0.002
			**B***	10.877	0.0150		-1.654	0.222
**Ev-mic control solutions**	0.625-10	5	**A***	2.8355	0.000287	0.9921	10.122	<0.001
			**B***	11.455	0.0199		0.933	0.356

* A, slope, B, intercept, r^2, fit correlation coefficients, t, Student's t statistics, p, p-value at 95% significance level.

Table S3- Evaluation of inter-day and intra-day precision and accuracy.

Concentration (µg/mL)	Intra-day (n=4)		Inter-day (n=4)	
	% Mean accuracy	% C.V.*	% Mean accuracy	% C.V.*
**Standard solutions**				
5	97.3	2.7	93.3	4.3
10	93.5	2.6	98.0	3.5
**Ev-sol control solutions**				
5	95.7	2.0	93.2	5.3
10	91.6	1.9	103.8	2.9
**Ev-mic control solutions**				
5	91.2	1.0	95.2	5.6
10	97.8	2.5	99.1	2.5

*C.V.= coefficient of variation

Table S4- Recovery determined for standard and Ev-sol and Ev-mic working solutions.

Concentration (µg/mL)	Relative recovery ± S.D. (peak area ratios, n=3)		
	standard	Ev-sol/standard	Ev-mic/standard
5	98.8± 1.5	99.4±2.6	100.3±3.2
10	99.3±2.8	100.6±1.9	98.8±2.1

	Absolute recovery± S.D. (peak area, n=3)	
	Ev-sol	Ev-mic
5	99.4±1.8	97.5±2.2
10	101.7± 2.1	100.2±1.6
